# Extracellular Vesicles Derived from Mesenchymal Stromal Cells Reduce *Pseudomonas aeruginosa* Lung Infection and Inflammation in Mice

**DOI:** 10.1101/2025.03.30.646208

**Published:** 2025-03-31

**Authors:** Sharanya Sarkar, Roxanna Barnaby, Zachary Faber, Lily Taub, Carolyn Roche, Brad Vietje, Douglas J. Taatjes, Matthew J. Wargo, Daniel J. Weiss, Tracey L. Bonfield, Thomas J. Kelley, Bruce A. Stanton

## Abstract

The World Health Organization and the U.S. Centers for Disease Control and Prevention have reported that antibiotic resistant infections with *Pseudomonas aeruginosa* present a significant health risk world-wide. In the genetic disease Cystic Fibrosis (CF), chronic antibiotic resistant *Pseudomonas* lung infections and persistent inflammation remain the leading causes of morbidity and mortality. While highly effective modulator therapy (HEMT) dramatically improves lung function in CF, they fail to eradicate chronic infections or eliminate the associated hyperinflammatory state. Thus, there is an urgent need for innovative therapies that can simultaneously eliminate antibiotic resistant *P. aeruginosa* lung infection and the attendant hyperinflammatory lung environment. Mesenchymal stromal cell-derived extracellular vesicles (MSC EVs) represent a promising solution, offering potent anti-inflammatory, immunomodulatory, and antimicrobial properties while being safe and non-toxic. This study demonstrates using a CF mouse model of infection that MSC EVs reduce acute *P. aeruginosa* lung infection and inflammation. MSC EVs reduced *Pseudomonas* burden, immune cell infiltration, and pro-inflammatory cytokine levels. As the first investigation of MSC EVs in CF, this research underscores the dual effects of MSC EVs; mitigating inflammation and reducing bacterial burden. These findings mark an important advancement in antimicrobial therapy, addressing the unmet need for reducing antibiotic resistant infections and hyperinflammation for people with CF as well as the multitude of others with chronic, antibiotic resistant *P. aeruginosa* lung infections.

**GRAPHICAL ABSTRACT:**

**NEW AND NOTEWORTHY:**

This is the first study demonstrating the ability of Mesenchymal Stromal Cell Extracellular Vesicles (MSC EVs) to reduce *Pseudomonas aeruginosa* burden and inflammation in a CF mouse model of infection.

## KEYWORDS

Extracellular vesicles; Mesenchymal Stromal Cells; Inflammation; Cystic Fibrosis; *Pseudomonas aeruginosa*

## GRAPHICAL ABSTRACT

## NEW AND NOTEWORTHY

This is the first study demonstrating the ability of Mesenchymal Stromal Cell Extracellular Vesicles (MSC EVs) to reduce *Pseudomonas aeruginosa* burden and inflammation in a CF mouse model of infection.

## INTRODUCTION

The World Health Organization and the U.S. Center for Disease Control and Prevention have reported that antibiotic resistant infections with *Pseudomonas aeruginosa* (*P. aeruginosa*) present a significant health risk world-wide. *P. aeruginosa* is the fifth most common cause of hospital-acquired infections, and in the U.S. alone 32,600 hospitalized patients had antibiotic resistant *P. aeruginosa* infections in 2017 (1). More than 50% of adults with the genetic disease Cystic fibrosis (CF) are chronically infected with antibiotic resistant strains of *P. aeruginosa* (2, 3), which is the primary contributor to morbidity and mortality in people with CF (pwCF) (4, 5).
Chronic infection in CF initiates a sustained inflammatory response that fails to resolve the infection and leads to progressive lung damage (6). A significant advancement in CF research has been the development of CFTR modulators, which are small molecules designed to correct protein misfolding or enhance channel gating to facilitate apical chloride and bicarbonate secretion (2). While highly effective modulator therapy (HEMT) has shown promising results in improving lung function and reducing hospitalization rates in pwCF, it does not eradicate bacterial lung infections or mitigate the hyperinflammatory response to chronic *P. aeruginosa* infections (7, 8). Thus, there is a pressing need to develop more effective therapies to eliminate chronic antibiotic resistant *P. aeruginosa* lung infections.

Therapeutic anti-inflammatory medications have demonstrated efficacy in mitigating the decline in lung function associated with chronic *P. aeruginosa* infection, especially in pwCF. However, existing anti-inflammatory drugs elicit liver, pancreas, and kidney complications rendering them inadequate as therapeutics for sustained inflammation associated with chronic infection (9).

Consequently, there is also a need for novel treatments to address inflammation in people with chronic *P. aeruginosa* infections. Given the substantial global burden of bacterial antimicrobial resistance (estimated to reach US$1 trillion/year by 2050), developing new antibacterial and anti-inflammatory therapies holds promise for addressing various respiratory infections beyond pwCF (10).

Mesenchymal stromal cells (MSCs), precursor cells originating from bone marrow, adipose tissue, or cord blood, possess immunomodulatory and antibacterial characteristics and have been investigated for various acute and chronic inflammatory lung conditions (11–14). MSCs exhibit low immunogenicity, allowing for their utilization as an allogeneic cell source without the risk of rejection (15). Additionally, MSCs demonstrate favorable anti-inflammatory and tissue regenerative properties, (16–18). MSCs themselves and the soluble factors they secrete impede the growth of *P. aeruginosa*, *Staphylococcus aureus*, and *Streptococcus pneumoniae* due to the secretion of antimicrobial peptides (19). These desirable attributes have rendered MSCs a subject of great interest to researchers, with ongoing investigations spanning over 1,200 clinical trials (11). The established safety and lack of toxicity of MSCs have led to regulatory approvals for MSC-based therapies in several countries (20). Efforts in the CF setting have demonstrated the safety of allogeneic human MSCs in a phase 1 study involving 15 adult pwCF (21).

Researchers initially believed that MSCs exerted their effects solely through direct cellular interactions (22) until two seminal papers in the field unveiled that extracellular vesicles (EVs) released by MSCs also possess the properties of their parent cells (23, 24). MSC-derived EVs contain an array of biomolecules, including proteins, mRNA, lipids, DNAs, and miRNAs (25), and have demonstrated paracrine effects in various animal models of injury as extensively reviewed elsewhere (22, 26–28). Additionally, MSC EVs have shown promise in mitigating inflammation and tissue damage associated with pulmonary diseases as comprehensively discussed in recent reviews (25, 29). Beyond their anti-inflammatory roles, MSC EVs exhibit antimicrobial properties (30, 31). MSC EVs offer several advantages over MSCs themselves. First, EVs are less prone to inflammatory environmental alterations than MSCs (32). Second, their nano-scale size allows them to traverse mucus layers and penetrate antibiotic resistant bacterial biofilms effectively (33, 34). Third, their smaller size and reduced expression of MHC molecules make them less susceptible to immune rejection compared to their parent cells (35). The multitude of these benefits, coupled with their proven safety in clinical trials, position MSC EVs as promising candidates for therapeutic intervention (36, 37). We have shown previously that the miRNA let-7b-5p in EVs secreted by primary human bronchial epithelial cells (HBECs), when combined with sub-minimum inhibitory concentrations (MIC) of aztreonam or carbenicillin, effectively eradicated the formation of antibiotic-resistant *P. aeruginosa* biofilms *in vitro* (38). This combination also increased the antibiotic susceptibility of both mucoid and non-mucoid clinical strains of *P. aeruginosa* (38).

Because MSC EVs contain negligible levels of let-7b, and let-7b has antibacterial and anti-inflammatory properties, the goal of this study was to engineer MSC EVs to contain let-7b-5p as a therapeutic approach to reduce infection and inflammation using CF mice as a model of infection (39, 40). To our knowledge there are no studies or registered clinical trials involving let-7 family members to treat antibiotic resistant *P. aeruginosa* lung infections (41), highlighting a significant knowledge gap.

Our data demonstrates that MSC EVs, even without antibiotics, decrease the burden of a clinical mucoid isolate of *P. aeruginosa* and immune cells and modulate cytokine levels in the lungs of CF mice, thereby highlighting their potential as a novel anti-infective and anti-inflammatory candidate for antibiotic resistant *P. aeruginosa* lung infections.

## MATERIALS AND METHODS

### Isolation of MSC-derived extracellular vesicles (MSC EVs) and miRNA transfection of MSCs

Human Bone Marrow-Derived MSCs were obtained from the ATCC (Cat # PCS-500-012). MSCs were cultured according to the manufacturer's instructions using the ATCC media (2% FBS: up to passage 4). MSCs were transfected with a miRNA mimic negative control (NC, that has no known targets in mice or *P. aeruginosa*) or miRNA let-7b-5p, obtained from Ambion (Austin TX) using Lipofectamine RNAiMAX Transfection Reagent (Invitrogen, Waltham, MA). Two days before transfection, MSC were seeded at $1x10^6$ per T-75 flask. Once ~80% confluent, the media was switched to supplement-free media, and cells were transfected with a 20 µM stock of let-7b-5p (Ambion catalog #4464066) or the negative control miRNA (Ambion catalog #4464058). A mixture of RNAiMax (1:1000 vol/vol of reagent to total cell media), OptiMem (Invitrogen catalog #31985070), and miRNA was made and allowed to sit for 5 minutes and then added dropwise to the flasks. One flask of MSCs was not transfected at all (referred to as control or untreated MSCs). The media were harvested one day after the transfection. EVs were purified from the MSC culture supernatants using the ExoQuick-TC EV isolation kit (#EXOTC50A-1, Systems Biosciences Palo Alto, CA). Briefly, suspended cells were removed from MSC culture supernatants by centrifugation at 4400 rpm for 15 min, and 12 mL of supernatants were concentrated to ~200 – 400 µL with a 30 kDa Amicon Ultra Centrifugal Filter (Millipore, Billerica, MA). The concentrate was mixed with 10 µL of ExoQuick-TC polymer per 50 µL concentrated media. Following overnight incubation at 4°C, EVs were isolated by centrifugation (1,500 g, 30 min) and resuspended in a final volume of 150 µL PBS. MSC growth media not conditioned by cells was subjected to the same isolation process and was termed process control (PC) to account for any effect of the cell culture media and possible contamination that may be introduced during the EV isolation process.

### qRT-PCR to assess let-7b-5p loading in MSC EVs

RNA was isolated from EVs (10 µL total EV volume) using the miRNeasy Mini Kit (Qiagen, catalog #1038703) according to the manufacturer's instructions and eluted in RNase-free water. cDNA was made using a Taqman miRNA Kit (Invitrogen, catalog #4366597). let7b-5p specific primers were obtained from Invitrogen (catalog #4395446). In preliminary studies U6 was selected as an appropriate reference gene for qRT-PCR, since U6 (Invitrogen, catalog #4398988) was not affected by experimental treatments.

**Characterization of MSC EVs**

MSC EVs were characterized via Exosome Analytical Services provided by RoosterBio Inc.; (Frederick, MD). Sample identity was blinded. Briefly, Nanosight NS300 was utilized to determine particle concentration and size. The following settings were used: 532 nm wavelength Laser, Camera Level: 14, Detection Threshold: 7, Focus: variable, and Screen Gain: 10 at analysis. Three 30-second videos were then analyzed using the Nanosight NS300 software. Protein concentration was measured using a BCA assay. Total lipid content was assessed using the MEMGlow 488, a fluorescent dye binding to extracellular vesicles' lipid bilayer. A standard curve was generated in the background matrix of the samples and used to quantify the lipid concentration in the sample. Total miRNA content was isolated from samples using the commercially available RNeasy Mini Kit to isolate RNA, and the miRNA concentration was then determined using the 2100 Bioanalyzer and the Small RNAKit. For determining EV purity, EVs were stained with the MEMGlow 488 membrane dye, and then stained and unstained particles of aliquots of the same sample were run on Nanoparticle Tracking Analysis (NTA) software using fluorescence detection and standard particle concentration analysis. Ratios of the particle concentrations of the stained and unstained samples were used to quantify the percentage of particles that stained positive for lipids (i.e., EVs). For the EV marker characterizations, CD81 was detected using capillary western blot. Protein measurements obtained using microBCA were used to load samples and run on Simple Jess. ALIX and albumin concentrations were measured using commercial ELISA kits.

**miRNA-seq analysis of MSC EVs**

Studies were also conducted to examine the miRNA content of MSC EVs by smRNA-seq, with the goal to determine the effect of transfection on the miRNA content of MSC EVs and to identify other miRNAs in MSC EVs that may have gene targets in *P. aeruginosa* or mouse cells. RNA was isolated from 10 µL of either control MSC EVs (no transfection), negative control miRNA transfected MSC EVs (NC MSC EVs) or let-7b-5p transfected MSC EVs (let-7b MSC EVs) using the miRNeasy Kit (Qiagen, Germantown, MD, Catalog #217004). In some cases RNA concentrations were too low to quantify, thus, the entire volume of extracted RNA was desiccated to 5 µL and used as input into the Qiaseq smRNA library preparation kit (Qiagen). For all samples, the workflow was performed according to manufacturer's instructions, using 24 cycles for final library PCR. Blinded samples were uniquely indexed and pooled for sequencing on a Nextseq200 instrument (Illumina), targeting 10 million reads per sample.

miRNA-Seq reads were first trimmed using CutAdapt v4.0 with trimming parameters "-m 1, --nextseq-trim=30, -q 30, --max-n 0.8". Unique molecular identifiers (UMIs) were then extracted from trimmed reads and appended to read named using UMI-tools extract (v1.1.5). Reads were aligned to human miRNA sequences from miRbase using Bowtie2 v2.4.4 with mapping parameters "-D 20, -R 3, -N 1, -L 12, -i, S,1,0.50". miRbase sequences were padded by 5 base pairs at both ends with native hg38 (GRCh38.97) sequences to maximize alignment rates. Mapped reads were filtered with Samtools v1.15.1 such that matches were required to be between sixteen and twenty-eight base pairs in length, with no gaps, less than one mismatches, and a MAPQ score ≥ 20. Remaining reads were deduplicated based on UMI sequences with UMI-tools dedup (--method unique). Reads aligning to miRbase were counted using Samtoolsidxstats to estimate expression levels. To facilitate detection of RNAs absent in miRbase, unmapped reads were aligned to the hg38 genome using Bowtie2 in very-sensitive-local mode, depulicated, and filtered following the same criteria above, and assigned to genes using featureCounts (Subread v2.0.1). Sequencing and alignment quality control metrics were assessed using Samtools v1.15.1, FastQC v0.11.9, and Multiqc v1.12.

miRNA $\log_2$ fold changes ($\log_2$FC) between all pairwise combinations of MSC EV samples (control MSC EVs, negative control transfected MSC EVs, and let-7b-5p MSC EVs) were calculated from TMM normalized counts per million (CPM), generated from the miRbase aligned miRNA raw counts using the edgeR package (version 4.2.2) in R version 4.4.0. We used the Rocket-miR program (42) to identify pathways in *P. aeruginosa* and other ESKAPE pathogens that are potentially targeted by miRNAs of interest. Rocket-miR uses the IntaRNA program to predict interactions between human miRNAs and bacterial proteins (42–44).

IntaRNA was also used to predict human and mouse gene targets (with human genome assembly GRCh38.p14 and mouse genome assembly GRCm39) for miRNAs that had absolute $\log_2$FC values greater than 1 in both the let-7b MSC EV and the NC MSC EV compared to control MSC EV. IntaRNA was run in IntaRNAsTar mode to optimize for whole genome searches. The top 10 gene targets by predicted interaction energy score were analyzed for functional relevance with DAVID (45, 46). We specifically focused on pathways and processes related to inflammation in both mice and humans.

**Electron microscopy of MSC EVs**

EVs were visualized with negative staining transmission electron microscopy. A 10-microliter aliquot of each sample was applied onto a freshly glow-discharged 200-mesh nickel grid and allowed to sit for 2 minutes. After this incubation, excess fluid was gently removed with filter paper, and the grids were sequentially rinsed on seven drops of Millipore-filtered distilled water. While the grids remained slightly damp, they were placed onto a drop of Nano-W tungsten stain (Ted Pella; Product No. 09S432, Lot No. 14702). Excess stain was immediately absorbed with filter paper, and the staining step was repeated on a second drop of Nano-W, which was allowed to sit for 1 minute before being wicked away and left to dry. Imaging of the stained grids was conducted at 80 kV on a JEOL 1400 transmission electron microscope (JEOL Inc., Danvers, MA), and TIFF-format images were captured using an AMT XR11 digital camera (AMT, Woburn, MA). For diameter measurements, the electron microscopy images were processed in MetaMorph Offline image analysis software (v.7.8.0.0; Molecular Devices LLC, San Jose, CA), where 25 EV diameters were measured by drawing a line across each vesicle. The average diameter was subsequently calculated. The imaging and measurements of the EV diameters were conducted by an investigator blinded to the sample identities.

**Preparation of agarose-containing beads**

Agarose-containing beads for embedding *Pseudomonas aeruginosa* were prepared as previously described (47). In summary, five colonies of a clinical, mucoid isolate of *P. aeruginosa* tagged with mCherry (mCH PA M57-15), grown overnight on gentamicin/LB agar, were inoculated into 25 mL of LB broth and incubated at 37°C with shaking at 200 rpm for 20 hours. The culture was then diluted tenfold to achieve an optical density (OD) at 600 nm of approximately 0.3. 23 mL of the mCH PA M57-15/agarose/PBS mixture was added to 250 mL of sterile mineral oil in a beaker and stirred on medium-high speed. After 10 minutes, the bead/oil mixture was transferred to 15 mL of prewarmed 0.5% sodium deoxycholate/PBS and centrifuged to separate the beads from the oil. After a second wash, the beads were resuspended in 20 mL of PBS, washed four times, and imaged under the red fluorescence channel. The bead/PBS mixture was then diluted to achieve a concentration of 25,000 CFU in 50 µL, as determined by plating on gentamicin/LB agar and colony-forming unit (CFU) enumeration.

### Inoculation of *Pseudomonas aeruginosa* in Mice

All experiments were conducted using gut-corrected F508del/F508del Cftr$^{tm1Kth}$ TG(FABPCFTR)1Jaw/CWR mice (C57BL/6 genetic background) that expressed CFTR in gut cells under the FABP promoter to minimize gut obstruction and enhance breeding efficiency (47). Institutional Animal Care and Use Committee (IACUC) approval from Case Western Reserve University was obtained for all protocols. Male and female mice aged 10–12 weeks were inoculated with agar beads loaded with $10^5$ CFU of the mCH PA M57-15 strain and $10^8$ MSC EVs or process control. Human-derived EVs have previously been evaluated in mouse models for cytotoxicity and immunogenicity, demonstrating safety (48) and efficacy across various experimental models (49, 50). However, administration of human-derived EVs are likely to elicit an allogenic immune response, whereas MSC EVs do not elicit an immune response. The inoculum was administered to the right lung using a micro-endoscopy system (Polydiagnost, Pfaffenhofen, Germany) because preliminary experiments indicated this method increased inoculation efficiency and reduced variability. Mice were placed on warming pads post-anesthesia with isoflurane and monitored until fully recovered. Daily assessments of the inoculated mice were conducted up to the day of sample collection, following established protocols (51). Three days post-infection, the mice were euthanized via $CO_2$ inhalation. After disinfecting with 70% ethanol, a midline incision was made to expose the thoracic cavity, and the diaphragm was incised to access the lungs. Bronchoalveolar lavage fluid (BALF) was collected, and both lobes of the lung were individually homogenized and cultured to assess *P. aeruginosa* growth (reported in CFU/mL). Total bacterial burden was calculated by summing colony forming units (CFU) counts from BALF, left lung, and right lung homogenates. Total white blood cells in BALF were quantified using a hemocytometer, and differential cell counts were determined through Giemsa/Miller staining. Serum and BALF cytokines were analyzed on all mice at euthanasia (day 3), using a Luminex 32-plex multianalyte cytokine array (Millipore Mouse 32-Plex Kit, EMD Millipore Corporation, Billerica, MA). All experimental protocols will be deposited in EV-TRACK to enhance transparency and interpretation of EV experiments and to increase experimental reproducibility.

### Statistical Analysis

To minimize potential bias, all analytic assays, mouse experiments and data analyses were performed by personnel blinded to the treatment. Statistical analysis was performed using GraphPad Prism (v10.3.1, San Diego, CA) and R (v 4.4.0) (52). Some mice with zero CFU counts were excluded from the analysis due to liquid handling errors. For count data such as CFUs and immune cells, negative binomial generalized Linear Models (glm.nb) were employed to compare control and EV-exposed groups. This approach was chosen because it is a robust and less biased method that appropriately accounts for the characteristics of bacterial count data, which are strictly non-negative and tend to be skewed toward larger values (53). Immune cell counts were analyzed using batch as a fixed effect, while batch was treated as a random effect for CFU counts to facilitate model convergence. For cytokine levels representing continuous data rather than counts, linear models were applied with batch included as a fixed effect. Statistical analyses were conducted using the *MASS* and *lme4* packages in R, with P-values derived accordingly. This methodological framework ensured accurate and reliable comparisons across experimental groups while accounting for batch variability.

The graphical abstract was created using the Premium version of BioRender with the following publication license: Sarkar, S. (2025) https://BioRender.com/p72w212

## RESULTS

### Isolation and characterization of MSC EVs

In alignment with the International Society of Extracellular Vesicles' (ISEV) objective to enhance the robustness of EV research (54), we conducted a comprehensive analysis of bone marrow MSC EVs (MSCs purchased from ATCC®, see methods). EVs were isolated from the conditioned media using ExoQuick-TC. Table 1 presents data on the characterization of MSC EVs, including the total protein, lipid, and miRNA content, as well as particle concentration, purity, and size. EV purity was assessed by determining the percent of particles that were labeled with a membrane dye and mean particle size was determined by Nanoparticle Tracking Analysis (NTA). The data presented in Table 1 all fall within the range of values for EVs specified by MISEV (54).

**Table 1: Characterization of MSC EVs.**

| Total protein ± SD (µg/mL) | Total lipid ± SD (µg/mL) | Total miRNA ± SD (pg/µL) | Particle concentration (particles/mL) | EV purity (%) | Mean particle size ± SD (nm) |
|---|---|---|---|---|---|
| 24.7 ± 1.2 | 14.6 ± 0.4 | 147.9 ± 32.5 | $6.8 \times 10^9$ | 46.4 | 151.1 ± 64.4 |

### Protein marker characterization and let-7b-5p loading of MSC EVs

To confirm that we isolated EVs, surface marker characterization was conducted according to guidelines set forth by MISEV (54). The guidelines state that EVs should exhibit the presence of at least one category 1 protein, one category 2 protein, and the absence of at least one category 3 protein. Our analysis via capillary western blot revealed the presence of CD81, a category 1 marker, and analysis via ELISA confirmed the presence of ALIX, a category 2 marker while indicating the absence of contaminating protein albumin, a category 3 marker **(Figure 1A)**. Since naïve MSC EVs do not contain detectable levels of let-7b-5p, we harvested EVs from control (untreated) MSCs and MSCs transfected with either a negative control (NC) miRNA (a miRNA with no known targets in humans, mice, or *Pseudomonas*) or let-7b-5p. To ensure successful let-7b-5p loading into MSC EVs, qRT-PCR was performed on EVs using let-7b-5p specific primers, with U6 as a reference gene. The results demonstrated that while U6 levels stayed consistent among the three groups ($\Delta$CT = 0.3), the CT (cycle threshold) value for let-7b-5p in EVs isolated from let-7b-5p transfected MSCs was lower than that from control or NC MSCs ($\Delta$CT = 8.65).

**(Figure 1B)**. This analysis demonstrated that our transfection protocol successfully loaded let-7b-5p into MSC EVs.

**A)**

| CD81 | ALIX ± SD (ng/mL) |
|---|---|
|   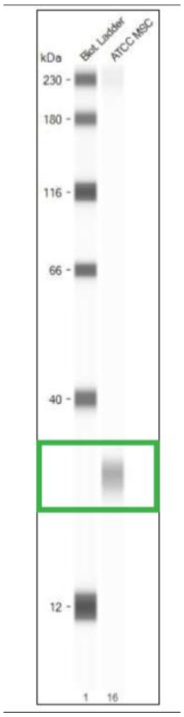 | 0.59 ± 0.04 |
| | **Albumin ± SD (ng/mL)** |
| | 0.00 ± 0.00 |

**B)**

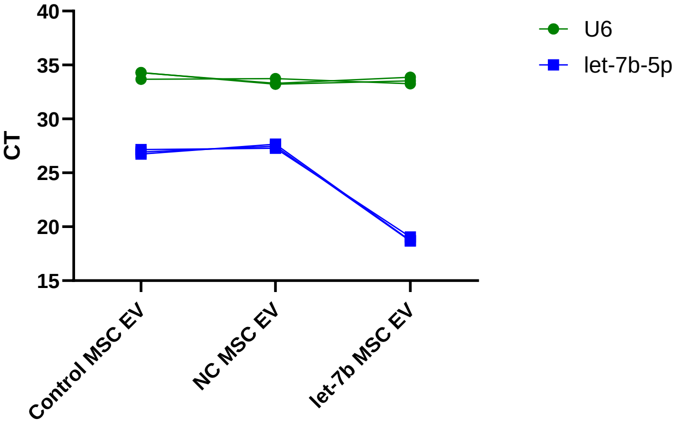

**Figure 1: A)** Protein marker characterization of MSC EVs. Capillary western blot confirmed the presence of tetraspanin CD81, while ELISA demonstrated the presence of cytosolic ALIX and the absence of albumin, a common contaminant in EV preparations. **B)** qRT-PCR assays revealed a significant decrease (ΔCT = 8.65) of let-7b-5p in EVs derived from let-7b-5p transfected MSCs compared to those from control MSCs or MSCs transfected with NC miRNA indicating successful let-7b-5p loading via parent cell transfection. The reference gene, U6, was not significantly affected by transfection (ΔCT = 0.3) among the three groups. n = 3 EV preparations; mean ± SEM depicted; NC = negative control.

### Visualizing MSC EVs with Electron Microscopy

We employed high-resolution negative staining transmission electron microscopy to visualize the EVs. Low magnification **(Figure 2A)** and high magnification **(Figure 2B)** images of the EV samples revealed the presence of vesicles. The EVs had an average diameter of 57.5 nm (n=25), well within the reported size range for EVs (55). On the other hand, process control or PC (which is unconditioned MSC growth media passed through the EV isolation process) lacked any identifiable vesicular structures, affirming that the MSC growth media did not contain vesicles. **(Figures 2C - 2D)**. Notably, there was a clear difference in the diameter of particles measured by Nanoparticle Tracking Analysis (151.1 nm) and negative staining electron microscopy (57.5 nm).

This is not surprising given previous examples in the literature of differences in EV size measured by NTA and electron microscopy (56, 57). NTA measures light scattering and provides hydrodynamic radii, thereby overestimating EV diameter (58). The fixation process for electron microscopy can shrink vesicles and reduce their diameter (59).

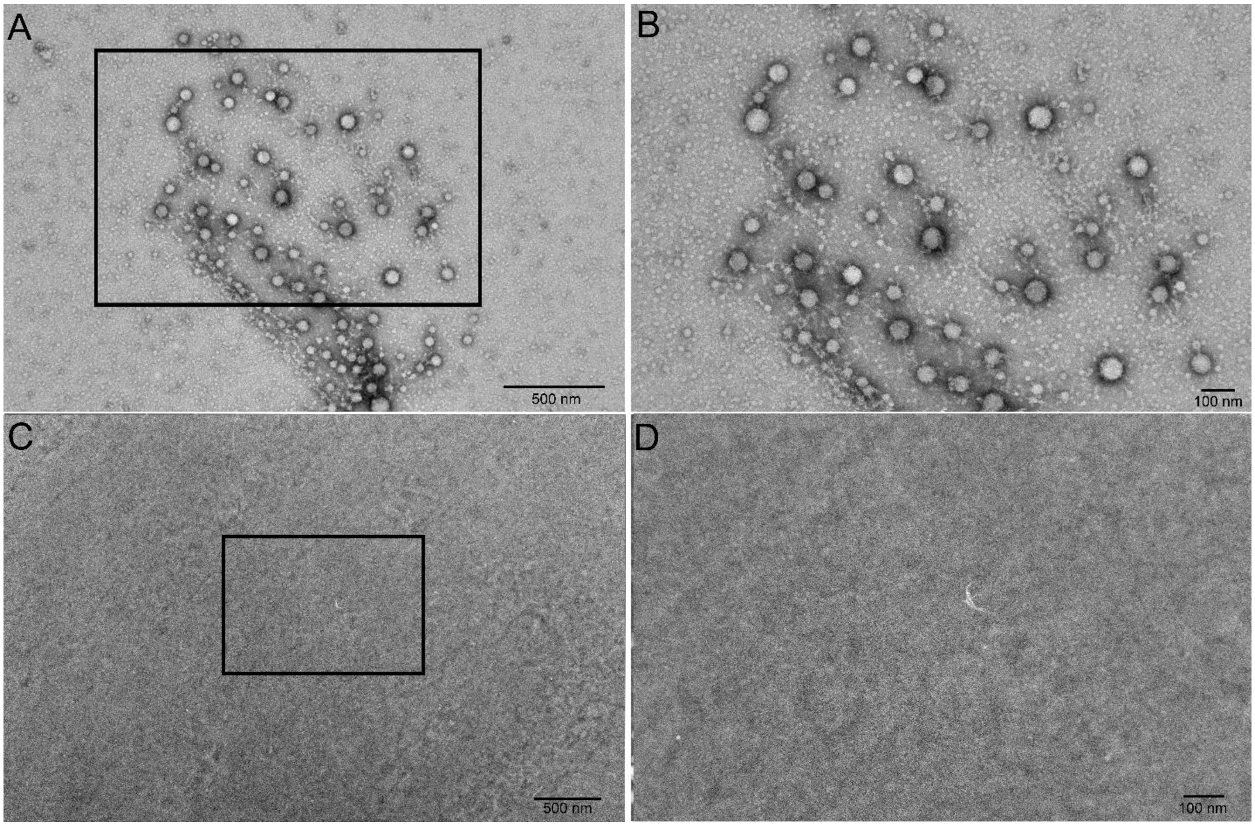

**Figure 2**: Transmission electron micrographs demonstrating the presence of MSC EVs under: **A)** low magnification (x6000), and **B)** high magnification (x50000). Transmission electron micrographs showing the lack of identifiable vesicles in the process control (unconditioned media run through EV isolation process) under **C)** low magnification (x6000) and **D)** high magnification (x50000). The boxes in A and C indicate the regions presented in B and D. Scale bars represent 500 nm and 100 nm for the low- and high magnifications, respectively.

**MSC EVs reduce the total *Pseudomonas* burden in CF mice**

To evaluate the effects of MSC EVs on *P. aeruginosa* infection and the immune response, CF mice were infected with *P. aeruginosa*-impregnated agarose beads along with either Process Control (PC), MSC EVs loaded with negative control miRNA (NC MSC EV) or MSC EVs loaded with let-7b-5p (let7b MSC EV). After 3 days, *P. aeruginosa* CFU/mL from the harvested lung tissue and BALF were measured to assess the total bacterial burden. Both NC MSC EVs and let-7b MSC EVs significantly reduced *P. aeruginosa* CFUs/mL compared to PC **(Figure 3)**. Notably, the reduction in CFUs was similar in mice exposed to NC MSC EVs and let-7b MSC EVs **(Figure 3)**.

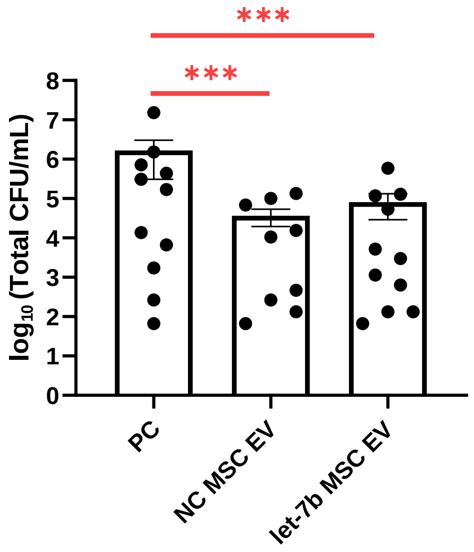

**Figure 3:** MSC EVs loaded with negative control (NC) miRNA or let-7b-5p reduced total lung *Pseudomonas aeruginosa* counts (CFU/mL) in CF mice by 97.8% and 95.1%, respectively, as compared with the process control (PC) group. Negative binomial generalized linear models were used to account for potential batch effects and to test the statistical significance between the PC and EV groups. Batch was modeled as a random effect to enable model convergence. Each data point represents one CF mouse. ***$P < 0.001$; $n$ = 9 - 11 mice/group; mean ± SEM depicted. Data were compiled from 3 independent experiments. CFU, colony-forming units; PC = process control (unconditioned medium passed through the EV isolation process).

**MSC EVs modulate two key cytokines in the BALF and serum of CF mice**

The effect of MSC EVs on inflammation was assessed by measuring the levels of cytokines in BALF and serum using Luminex multi-plex technology. NC MSC EVs significantly increased IL-10 in BALF, compared to PC. Although the let-7b MSC EVs tended to increase IL-10 compared to PC, the change was not significant **(Figure 4A)**. In serum, NC MSC EVs significantly reduced the levels of the pro-inflammatory cytokine RANTES compared to PC, while the let-7b MSC EV group showed a tendency to decrease RANTES but the change was not significant **(Figure 4B)**. IL-10 is an anti-inflammatory cytokine that can limit tissue damage to the host (60), a process that will be of utmost importance to people chronically infected with *P. aeruginosa.* The reduction in RANTES will reduce migration of lymphocytes and monocytes to the lungs, which would be beneficial to pwCF since recruitment of CF lymphocytes and monocytes in CF is excessive and prolonged, thereby leading to tissue damage (61). The differential change of cytokines in BALF versus serum is likely due to different levels of cytokine-binding proteins, and soluble receptors for cytokines in the two different biological fluids (62). These results demonstrate that MSC EVs, like MSCs, are anti-inflammatory and pro-resolving. Taken together, the data suggest that MSC EVs modulate cytokines in a way that would be beneficial to pwCF.

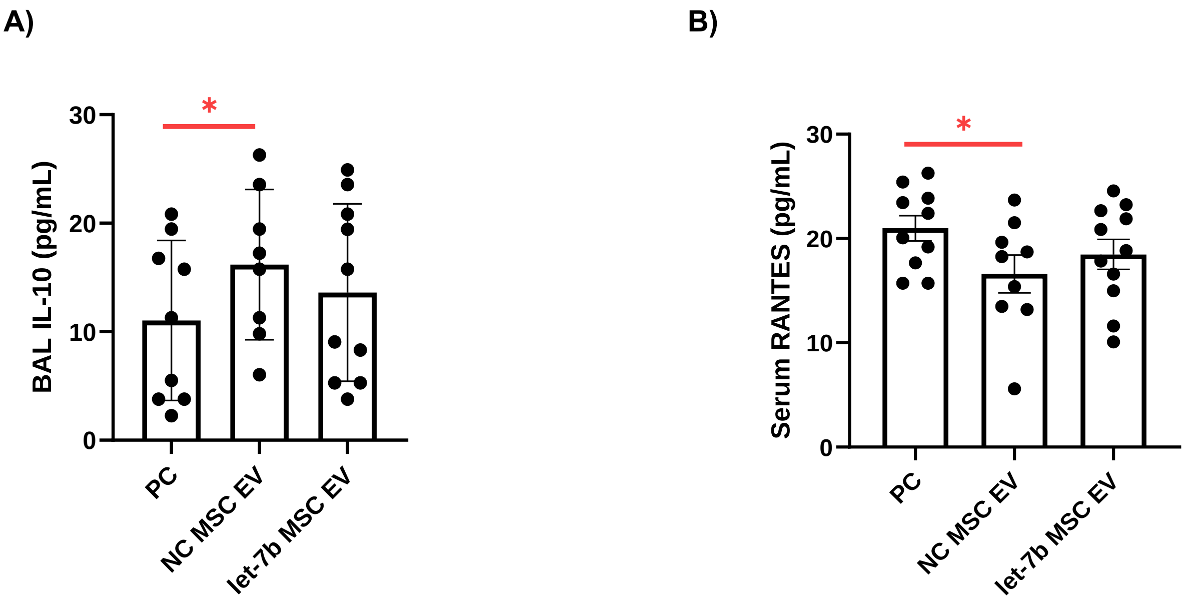

**Figure 4:** MSC EVs engineered to contain negative control (NC) miRNA significantly increased **A)** BALF IL-10 (by 46.4%) and decreased **B)** Serum RANTES (by 20.8%) as compared to the process control (PC) group. let-7b-5p loaded MSC EVs tended to increase IL-10 in BALF (by 23.2%) and decrease serum RANTES (by 11.9%) with respect to control mice. Linear models with batch as a fixed effect were used to account for potential batch effects and test the statistical significance between the control and EV groups. Each data point represents one CF mouse. Mice which had IL-10 and RANTES levels beyond the detection level of the multiplex platform (0.1 -1 pg/mL) were excluded from the analyses. $^*P < 0.05$; $n$ = 8 - 11 mice/group; mean ± SEM depicted. Data were compiled from 3 independent experiments. A power analysis of the data (at p = 0.05 and power = 0.80) revealed that an additional 140 mice are needed to obtain statistically significant changes in IL-10 and an additional 48 mice are needed to obtain significant changes in RANTES in let-7b MSC EV compared to PC, making further mouse experiments impractical.

**MSC EVs reduce immune cell numbers in the BALF of CF mice**

Studies were also conducted to explore the effects of MSC EVs on immune cell counts in the BALF of infected CF mice. Compared to PC, NC MSC EVs significantly reduced total cells (a summation of monocytes, lymphocytes, and neutrophils) in BALF and let-7b MSC EVs demonstrated a very strong tendency (p-value = 0.06) to reduce total cell counts **(Figure 5A)**. We then looked at differential cells counts and found that both NC MSC EVs and let-7b MSC EVs significantly reduced lymphocytes **(Figure 5B)** and monocytes **(Figure 5C)** in BALF. Neutrophil counts decreased significantly with NC MSC EVs and tended to decrease with let-7b MSC EVs **(Figure 5D)**. Although not statistically significant (p-value = 0.13) the decreases in neutrophil counts was 39.9%, a decrease likely to have a positive effect since neutrophils in CF have a defect in the ability to kill *P. aeruginosa* and CF neutrophils release proteases and DNA that cause lung damage, increase mucus viscosity and thereby the ability to clear bacteria (63). The lack of significance in total cell reduction for let-7b MSC EVs is attributable to the limited effect on neutrophil counts. Notably, MSC EVs in this study outperformed Human Bronchial Epithelial Cell

EVs (HBEC EVs) reported in our previous study (47), as HBEC EVs failed to reduce total immune cell numbers following *P. aeruginosa* infection in CF mice.

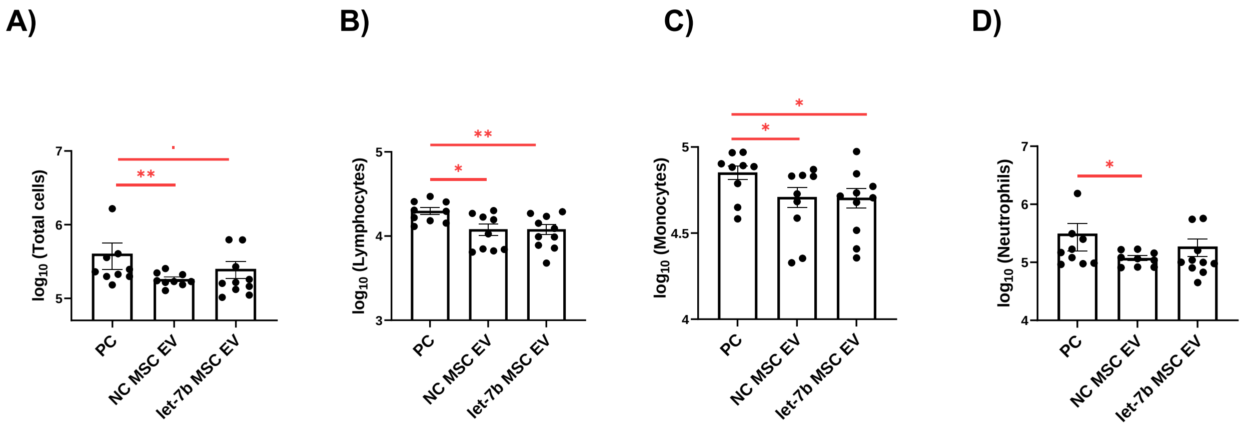

**Figure 5: A)** Total cell count in BALF was significantly reduced by NC MSC EVs (by 54.8%) but was not significantly reduced by let-7b MSC EVs (by 37.9%). MSC EVs loaded with negative control (NC) miRNA or let-7b-5p significantly decreased **B)** lymphocytes (39.4% by NC MSC EVs; 39.4% by let-7b MSC EVs) in the BALF of CF mice as compared to PC, and **C)** monocytes (27.9% by NC MSC EVs; 28.6% by let-7b MSC EVs). **D)** NC MSC EVs significantly reduced neutrophils (by 61.9%), whereas let-7b MSC EVs tended to reduce neutrophils, but the change was not significant (39.9%). The control group was exposed to process control (PC). Negative binomial generalized linear models with batch as a fixed effect were used to account for potential batch effects and test the statistical significance between the control and EV groups. Each data point represents one cystic fibrosis (CF) mouse. $*P < 0.05$, $**P < 0.01$; $n$ = 9-10 mice/group; mean ± SEM depicted. Data were compiled from 3 independent experiments. PC = process control. A power analysis of the data (at p = 0.05 and power = 0.80) revealed that an additional 83 mice are needed to obtain statistically significant changes in neutrophils in let-7b MSC EV compared to PC, making further mouse experiments impractical.

**Transfection of MSC with NC or let-7b-5p miRNAs altered the abundance of several miRNAs in EVs isolated from NC MSC and let-7b-5p MSC compared to EVs secreted by untreated MSC**

To determine why let-7b MSC EVs were not more effective that NC MSC EVs in altering cytokine secretion and immune cell counts, despite the well-known anti-bacterial and anti-inflammatory effect of let-7b, studies were conducted to interrogate the miRNA content of MSC EVs, NC MSC EVs and let-7b MSC EVs. miRNA-seq revealed that the transfection procedure altered the miRNA content of NC MSC EVs and let-7b MSC EVs compared to untreated MSC EVs. The 20 miRNAs with the largest (10) and smallest (10) $log_2FC$ values for the three different preparations of MSC EVs are presented in Figure 6 [NC MSC EVs vs. EVs isolated from untransfected MSCs (**Figure 6A**); let-7b MSC EVs vs. EVs isolated from untransfected MSCs (**Figure 6b**) and let-7b MSC EVs vs. NC MSC EVs (**Figure 6C**)]. The data reveal that the transfection process changed the levels of miRNAs in NC MSC EVs and let-7b MSC EVs compared to control MSC EVs. For instance, the abundance of some miRNAs either doubled ($log_2FC > 1$) or halved ($log_2FC < -1$) in the NC MSC EVs **(Figure 6A)** and let-7b MSC EVs compared to the EVs isolated from untransfected MSCs **(Figure 6B)**.

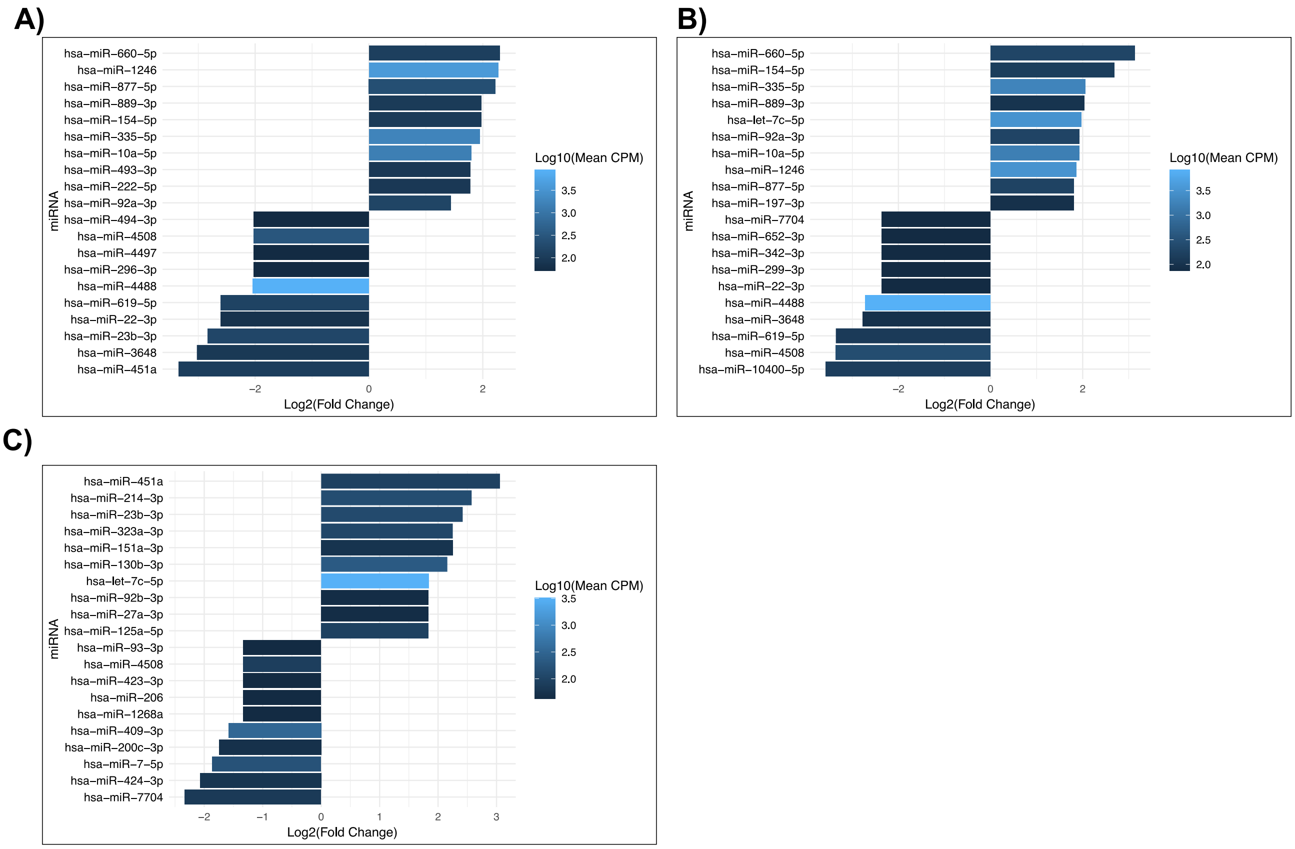

**Figure 6:** Comparison of miRNAs detected in: **A)** MSC EVs loaded with negative control (NC) miRNA vs. EVs isolated from untransfected MSCs; **B)** MSC EVs loaded with let-7b-5p miRNA vs. EVs isolated from untransfected MSCs and **C)** let-7b-5p loaded MSC EVs vs. NC loaded MSC EVs. The plots show the 20 miRNAs with the largest and smallest $log_2FC$ values for each comparison. Bar colors correspond to the mean counts per million calculated across the samples in each comparison. miRNA let-7b-5p was excluded from the analysis due to its large $log_2FC$ values that would dominate the graphs (see **Figure 7** for let-7b-5p data).

Using the data in Figure 6 we identified 8 miRNAs whose abundance increased by over 100% and 5 miRNAs whose abundance decreased by more than half in both the let-7b MSC EVs and NC MSC EVs compared to the EVs isolated from untransfected MSCs. Notably, miR-877-5p was elevated in both NC MSC EVs and let-7b MSC EVs. miRNA-877-5p and let-7b-5p have greater than 50% overlap in their potential *P. aeruginosa* gene targets, as determined by the Rocket-miR application that we developed to predict druggable targets by miRNAs in 24 species of bacteria (42). This observation may explain why we saw a similar reduction in *P. aeruginosa* burden for both the let-7b and NC MSC EV groups compared to PC in CF mice. **Table 2** highlights the top 10 KEGG pathways of predicted miR-877-5p and let-7b-5p gene targets for *P. aeruginosa* and other CF pathogens. The other pathogens such as *Staphylococcus aureus*, *Streptococcus*

*sanguinis*, and *Prevotella melaninogenica* were chosen because a data mining publication from our laboratory revealed that these bacteria accounted for 24% of the variability in lung function in a cohort of 167 pwCF (64). Pathways highlighted in red have greater than 50% of their genes predicted to be targets of let-7b-5p and miRNA-877-5p. These observations suggest that let-7b-5p would have important impacts on other bacteria, and that the upregulation of miRNA-877-5p in NC MSC EVs, induced by transfection, may account for the similar effects of NC MSC EVs and let-7b-MSC EVs.

**Table 2: KEGG pathways in CF pathogens with the highest percentage of genes targeted by miR-877-5p, with highlighted pathways indicating those in which over 50% of genes are also targeted by let-7b-5p.** Some of the genes in biofilm formation and antibiotic resistance pathways were identified previously by us as targeted by let-7b and miRNA-877-5p using Rocket-miR (42).

| ***Pseudomonas aeruginosa*** | ***Staphylococcus aureus*** | ***Streptococcus sanguinis*** | ***Prevotella melaninogenica*** |
|---|---|---|---|
| Acarbose and validamycin biosynthesis<br>Naphthalene degradation<br>Degradation of flavonoids<br>Viral life cycle<br>Non-homologous end-joining<br>Pentose and glucuronate interconversions<br>Nucleotide excision repair<br>Starch and sucrose metabolism<br>RNA polymerase<br>Alanine, aspartate and glutamate metabolism | Xylene degradation<br>Benzoate degradation<br>Monobactam biosynthesis<br>Degradation of aromatic compounds<br>Valine, leucine and isoleucine degradation<br>Teichoic acid biosynthesis<br>Glutathione metabolism<br>Lysine degradation<br>Tryptophan metabolism<br>Fatty acid metabolism | Glycan degradation<br>Valine, leucine and isoleucine degradation<br>Penicillin and cephalosporin biosynthesis<br>Carbapenem biosynthesis<br>Sphingolipid metabolism<br>Flagellar assembly<br>Sulfur relay system<br>Selenocompound metabolism<br>Glutathione metabolism<br>Streptomycin biosynthesis | Degradation of flavonoids<br>Biosynthesis of various plant secondary metabolites<br>Beta-lactam resistance<br>Oxidative phosphorylation<br>Nucleotide excision repair<br>Ubiquinone and other terpenoid quinone biosynthesis<br>Mismatch repair<br>Histidine metabolism<br>Flagellar assembly<br>Peptidoglycan biosynthesis |

Importantly, using IntaRNA and the DAVID functional annotation tool, we found that 16 miRNAs were differentially expressed and predicted to reduce inflammation in mice. For instance, miRNA-206 and miRNA-424-3p increased in NC EVs relative to let-7b-5p MSC EVs. Both miRNAs target genes involved in PI3K-Akt and MAPK proinflammatory signaling respectively. miRNA-451a and miRNA-125a-5p increased in let-7b MSC EVs compared to NC MSC EVs and target genes involved in B-cell activation and NF-κB signaling, respectively **(Table 3)**. Thus, both NC MSC EVs and let-7b-5p EVs contain different sets of anti-inflammatory miRNAs possibly providing an explanation as to why both sets of MSC EVs reduced inflammation.

Given our interest in using MSC EVs as a potential therapy, we investigated potential miRNA targets in human genes involved in inflammation. Our analysis revealed that several miRNAs differentially expressed in MSC EVs containing NC or let-7b-5p are also predicted to target key inflammatory pathways in humans. For example, miRNAs miR-206 and miR-93-3p enriched in NC EVs are predicted to suppress genes associated with NF-κB and JAK-STAT signaling. miRNAs miR-27a-3p and miR-92b-3p, which are more abundant in let-7b-5p MSC EVs are predicted to suppress genes involved in PI3K and MAPK pathways **(Table 4)**. This difference in the miRNA makeup may explain why NC MSC EVs and let-7b MSC EVs have slightly different effects on the secretion of some cytokines and immune cells in BALF, and is a topic for further investigation. In summary, the miRNA analysis identified several miRNAs that may be responsible for the observed reduction in *P. aeruginosa* burden as well as anti-inflammatory effects observed in CF mice treated with NC MSC EVs and let-7b-5p MSC-EVs compared to untransfected MSC EVs.

**Table 3: miRNAs in NC MSC EVs and let-7b-5p MSC EVs are predicted to have differential effects on several inflammatory pathways in mice.**

| **miRNAs upregulated in NC MSC EVs vs let-7b-5p MSC EVs** | | |
|---|---|---|
| ***miRNA*** | ***Gene target of miRNA*** | ***KEGG pathways related to inflammation (or Biological Process if KEGG pathway information was unavailable)*** |
| miR-93-3p | Ebag9 | T-cell mediated cytotoxicity |
| miR-200c-3p | Rictor | PI3K/protein kinase B pathway |
| miR-206 | Gng4 | PI3K-Akt pathway |
| miR-409-3p | Ahr | Th17 differentiation |
| miR-424-3p | Ppp5c | MAPK signaling |
| | Setx | MAPK cascade, PI3K/protein kinase B pathway |
| miR-1268a | Apbb1ip | T-cell activation |
| miR-4508 | Dysf | Cytokine production, macrophage activation, neutrophil chemotaxis |
| | Itga2b | PI3K-Akt pathway, Neutrophil extracellular trap formation |
| | Ntrk2 | MAPK signaling, PI3K-Akt pathway |
| | Tfr2 | TGF-β signaling |
| miR-7704 | Rapgef1 | PI3K/protein kinase B pathway, ERK cascade |
| | Hdac7 | Neutrophil extracellular trap formation |
| **miRNAs upregulated in let-7b MSC EV vs NC MSC EV** | | |
| ***miRNA*** | ***Gene target of miRNA*** | ***Examples of KEGG pathway of target gene related to inflammation (or Biological Process if KEGG pathway information was unavailable)*** |
| miR-let-7c-5p | Cacna2d2 | MAPK signaling |
| miR-mir-27a-3p | Ankrd26 | ERK cascade |
| miR-92b-3p | Taok2 | MAPK signaling |
| | Shc1 | Chemokine signaling pathway, NK cell mediated cytotoxicity |
| | Ttll12 | Innate immune response |
| miR-125a-5p | Ripor1 | Cellular response to chemokine |
| | Ift80 | NF-κB signaling, PI3K/protein kinase B pathway |
| miR-130b-3p | Stx7 | T-cell mediated cytotoxicity |
| miR-214-3p | AI182371 | Inflammatory response, complement activation |
| miR-323a-3p | Fbxw10 | Inflammatory response |
| miR-451a | Ms4a1 | B-cell activation |

**Table 4: miRNAs in NC MSC EVs and let-7b MSC EVs that are predicted to have differential effects on several inflammatory KEGG pathways in humans.**

| **miRNAs upregulated in NC MSC EVs vs let-7b MSC EVs** | | |
|---|---|---|
| ***miRNA*** | ***Gene target of miRNA*** | ***KEGG pathways of target gene related to inflammation (or Biological Process if KEGG pathway information was unavailable)*** |
| miR-93-3p | MYO18A | NF-κB transduction, macrophage activation |
| miR-206 | ZFP91 | NF-κB transduction |
| | SOCS1 | JAK STAT signaling |
| miR-409-3p | JUN | MAPK signaling, TLR signaling, IL-17 signaling, TNF signaling |
| miR-423-3p | CCDC88A | PI3K signaling |
| | MRAS | MAPK signaling |
| miR-4508 | CSPG4 | MAPK signaling |
| **miRNAs upregulated in let-7b MSC EV vs NC MSC EV** | | |
| ***miRNA*** | ***Gene target of miRNA*** | ***KEGG pathway of target gene related to inflammation (or Biological Process if KEGG pathway information was unavailable)*** |
| miR-let-7c-5p | CMKLR1 | Inflammatory response, macrophage chemotaxis, complement receptor mediated signaling |
| | LTBR4 | Inflammatory response, immune response |
| miR-23b-3p | TNFSF8 | Cytokine-cytokine receptor interaction |
| | DLL4 | T-cell differentiation |
| miR-27a-3p | PIK3C2A | PI3K pathway |
| miR-92b-3p | TAOK2 | MAPK signaling |
| | CDKN1A | JAK STAT signaling, PI3K-Akt pathway |
| miR-125a-5p | ZBTB33 | Cytokine production regulation |
| miR-130b-3p | RGMB | TGF-β signaling |
| miR-214-3p | HRNA4 | B-cell activation |
| | STAB1 | Inflammatory response |
| miR-323a-3p | CFLAR | NF-κB signaling, TNF signaling |
| miR-451a | NPNT | ERK cascade |

In addition, smRNA-seq validated the qPCR experiments (Figure 1B) demonstrating that transfection of MSC with let-7b-5p increased its abundance compared to NC MSC EVs and untransfected MSC **(Figure 7)**.

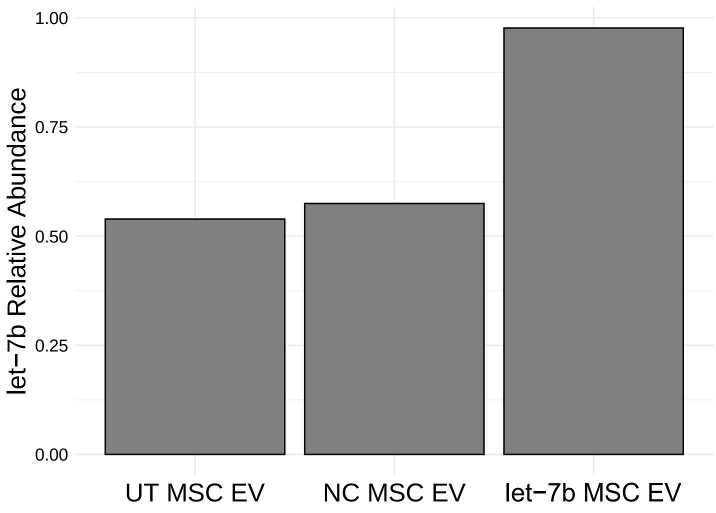

**Figure 7:** Relative abundance of let-7b-5p miRNA transcripts in EVs isolated from untransfected MSCs (UT MSC EV), EVs isolated from MSCs transfected with a NC miRNA (NC MSC EV) and EVs isolated from MSCs transfected with let-7b (let-7b MSC EV).

## DISCUSSION

Our mouse model of infection demonstrated that MSC EVs effectively reduced the *P. aeruginosa* burden and immune cell recruitment in the lungs while shifting the cytokine profile toward a more anti-inflammatory/pro-resolving landscape, even in the absence of antibiotics. MSC EVs increased IL-10, an anti-inflammatory and pro-resolving cytokine in BALF and decreased serum RANTES, a chemoattractant for immune cells. In addition, MSC EVs reduced monocytes, neutrophils and lymphocytes in BALF of CF mice. Our study is in general agreement with the studies of Shi et al (31) and extend the significance of MSC EVs to a frequently utilized CF mouse model of acute *P. aeruginosa* infection, which manifests excessive inflammation and a reduced ability to eliminate infections compared to non-CF mice (65). Shi et al. showed that MSC EVs significantly reduced mortality, neutrophil influx, proinflammatory factors, and bacterial burden in the BALF of non-CF mice with multidrug-resistant *Pseudomonas aeruginosa* pneumonia (31). Park et al. found that intravenous administration of MSC EVs effectively reduced bacterial burden in ex-vivo human lung tissue with severe bacterial pneumonia (30). The present study is also consistent with a previous report in which we demonstrated that EVs secreted by primary human airway epithelial cells endogenously containing let-7b-5p suppressed *P. aeruginosa* burden, reduced immune cells in BALF and decreased the proinflammatory response to *P. aeruginosa* even in the absence of antibiotics (47).

The inability of MSC EVs containing let-7b-5p to reduce bacterial burden compared to MSC EVs + NC was not surprising given our earlier *in vitro* study demonstrating that let-7b-5p in combination with sub minimum inhibitory concentrations of either aztreonam or carbenicillin was required to reduce *P. aeruginosa* (38). The combination of let-7b and aztreonam was required to reduce *P.*

*aeruginosa* due to the mechanism of action of let-7b-5p, which is to reduce aztreonam antibiotic efflux pumps, rendering aztreonam more effective in killing *P. aeruginosa* (38).
The mechanism of action of EVs is usually attributed to the release of their cargo into recipient cells. We have shown that HBEC EVs transfer the miRNA let-7b-5p to *Pseudomonas*, and blocking the miRNA via an anti-sense nucleotide to let-7b abolished the ability of let-7b plus aztreonam to reduce *P. aeruginosa* CFUs (38). Direct expression of let-7b-5p in *Pseudomonas* had a similar effect, indicating that let-7b-5p in combination with aztreonam reduces *Pseudomonas* CFUs. Thus, additional studies are required to evaluate the ability of MSC EVs and aztreonam together to reduce *P. aeruginosa* burden and reduce inflammation in CF mice.

Studies were also conducted to determine why NC MSC EVs and let-7b MSC EVs had many similar effects in mice. Analysis of the miRNA content of the MSC EVs revealed that transfection of a control miRNA as well as let-7b-5p in MSC changed the abundance of other miRNAs in EVs compared to untreated MSC. Most notably, transfection with the control miRNA increased the expression of miRNA-877-5p, which has similar predicted gene targets in *P. aeruginosa* as let-7b-5p. Examining the most prevalent miRNAs with predicted antibacterial properties, we propose that the effects of miRNA-877-5p (and possibly other miRNAs or other components in NC MSC EVs) may mediate the reduction of *P. aeruginosa* in mice exposed to NC MSC EVs, whereas let-7b-5p (and possibly other miRNAs or other components in let-7b MSC EVs) reduced *P. aeruginosa* in mice exposed to let-7b-5p MSC EVs. Elucidating the effects of the relative changes in numerous miRNAs, and possibly other components, in the MSC EVs on *P. aeruginosa,* immune cells and cytokine abundance is beyond the goals of this study and will require an intensive effort to unravel.

Our study has several notable strengths. First, the CF mouse model has been used extensively to study *P. aeruginosa* lung infections, thus, it is a well characterized and established model (51). Second, developing effective anti-Pseudomonal therapies is essential for all people with antibiotic resistant infections by *Pseudomonas*. The ability of MSC EVs to reduce bacterial burden and inflammation underscores their potential as a therapeutic option. MSC EVs are particularly compelling due to their dual mechanism of action: potent anti-bacterial and anti-inflammatory properties, the latter of which could help prevent infection-induced lung damage. Additionally, MSC EVs are known for their intrinsic pro-resolving, safe, and non-toxic characteristics, distinguishing them from conventional anti-inflammatory drugs that often have severe side effects. A Phase I clinical trial demonstrated the safety and non-toxic effects of parent MSCs in adults with CF (21), providing a strong foundation for exploring MSC EVs in pwCF. Our focus on the efficacy of engineered MSC EVs in mitigating bacterial burden, particularly against clinical mucoid isolates of *Pseudomonas aeruginosa*, is an important step forward. Third, the use of process control (unconditioned media subjected to EV isolation process) instead of saline or PBS as a control has several advantages, including minimizing potential factors in culture media or contaminants that may be added inadvertently in the isolation process that may affect infection and inflammation. Finally, MSC EVs have beneficial effects, including reducing inflammation, in several other disease models including Chronic Obstructive Pulmonary Disease (COPD), COVID-19, asthma, acute respiratory distress syndrome (ARDS), and idiopathic pulmonary fibrosis (IPF) (66), all via transfer of different miRNA/ protein factors into target cells. Thus, we predict that the use of MSC EVs engineered to contain anti-bacterial and anti-inflammatory miRNAs will be beneficial to numerous disease states characterized by antibiotic resistant bacteria and excessive inflammation. Moreover, we anticipate that our Rocket-miR program will facilitate identification of miRNAs that target genes involved in all antibiotic-resistant infections.

Despite its strengths, our study has some limitations. First, the agarose bead model in CF mice allows for only short-term infection studies (up to 10 days) and does not fully replicate the chronic bacterial infections seen in pwCF, although the bactericidal effects of antibiotics in mice frequently translate to studies in humans (6, 47, 51). Moreover, there was considerable variation in the response of mice in all experimental sets, which is not uncommon in experiments in mice, but still presents difficulties in detecting small changes in responses. Another limitation of this study is the simultaneous administration of MSC EVs and bacteria. Future studies will explore staggered treatments, where bacteria are introduced first, followed by MSC EVs. This approach would more closely mimic clinical scenarios where treatment is initiated after the onset of infection. In addition, although in a few assays there appeared to be an effect of let-7b MSC EVs compared to PC, the changes were not significant. We considered conducting additional studies to determine if the trend would be significant, however, a power analysis indicated that an additional 48 - 140 mice would be needed, which is not realistic. Finally, while this study focused on *Pseudomonas aeruginosa*, which colonizes approximately 50% of adults with CF, the CF lung microbiome is typically polymicrobial, including pathogens like *S. aureus*, *S. sanguinis*, *P. melaninogenica*, *Haemophilus influenzae*, and *Burkholderia cenocepacia* and the presence of other bacteria may affect the response of *P. aeruginosa* to MSC EVs, since the presence of other bacteria affect gene expression and antibiotic resistance of *P. aeruginosa* (67, 68). Our program Rocket-miR (42) predicts that let-7b-5p targets genes in other bacteria common in lung infections, including *P. melaninogenica*, *S. aureus*, and *S. sanguinis*. Future studies should explore the effects of let-7b-5p MSC EVs on polymicrobial infections to broaden the therapeutic scope. In addition, Rocket-miR also identified several other miRNAs that are predicted to target key genes in 24 bacterial genera, including all 6 ESKAPE pathogens, which are highly virulent and often antibiotic resistant.

In conclusion, our data demonstrates that MSC EVs reduce *P. aeruginosa* infection, immune cells and inflammation in our CF mouse model of infection. We hypothesize three potential mechanisms by which MSC EVs reduce bacterial burden and inflammation, none mutually exclusive. First, MSC EVs exhibit direct bactericidal activity, as shown in our previous studies (19). Second, MSC EVs may enhance phagocytosis and bacterial killing by CF macrophages and other immune cells, thereby reducing bacterial load. Third, MSC EVs may increase the secretion of antimicrobial peptides by airway epithelial cells. Future experiments will test these mechanisms to better understand how MSC EVs function *in vivo*. This knowledge will be instrumental in developing MSC EVs as dual-action anti-infective and anti-inflammatory therapy for all people with chronic infection and inflammation.

## ACKNOWLEDGEMENTS

This study was supported by the Cystic Fibrosis Foundation (STANTO19G0, STANTO20P0, STANTO23R0, and STANTO19R0) and the National Institutes of Health (P30-DK117469 and R01HL151385) to BAS. This work was supported by CFF RDP R447-CR11 and CFF

HODGES19R1 (Phenotyping Core). We thank RoosterBio for analysis of the MSC EVs (Table 1 and Figure 1A). smRNA-seq was carried out in the Genomics and Molecular Biology Shared Resource (RRID:SCR_021293) at Dartmouth which is supported by NCI Cancer Center Support Grant 5P30CA023108 and NIH S10 (1S10OD030242) awards. Support for RNA-seq data analysis was provided through Dartmouth Cancer Center Bioinformatics & Biostatistics Shared Resource (BBSR) supported by NCI award P30CA023108 and Dartmouth's Center for Quantitative Biology (CQB) Genomics Data Sciences Core (GDSC) supported by NIH award 3P20GM130454.

## DATA AVAILABILITY

Data will be made available upon reasonable request. EV sequencing data has been uploaded to NCBI GEO (Accession number: GSE290294). Detailed methods will also be uploaded to EV-track to enhance rigor and reproducibility.

## DISCLAIMERS

The funders had no role in study design, data collection and analysis, publication decisions, or manuscript preparation.

## DISCLOSURES

The authors declare no conflicts of interest, financial or otherwise.

## AUTHOR CONTRIBUTIONS

S.S., R.B., Z.F., M.W., D.J. W., T.L.B., T.J.K., and B.A.S. conceived and designed research; S.S., R.B., Z.F., C.R., B.V., performed experiments; S.S. and L.T. analyzed data; S.S., R.B., Z.F., L.T., T.J.K., and B.A.S. interpreted results of experiments; S.S. and L.T. and B.V. prepared figures; S.S. and B.A.S. drafted the manuscript; all authors edited and revised the manuscript; all authors approved the final version of the manuscript.
